# Survival and reactivation risk after heart transplantation in Chagas heart disease: a systematic review and individual patient data meta-analysis

**DOI:** 10.3389/fcvm.2026.1950776

**Published:** 2026-10-28

**Authors:** Zhi-hao Song, Guzinayi Abudukadi, Rong-Dong He, Aliya Tulading, Yilizhati Azhati, Maiwulanjiang Maimaiti, Mirizhati Nuermaimaiti, Chun-Hui Lv, Ming-Juan Wang, Hui-cheng Zeng, Guliziran Keranmu, Kurbanov Anvarbek Ikrom Ugli, Khimmatova Munisa, Tuerhongjiang Tuxun, Hao Wen

**Affiliations:** 1 Department of Liver Transplantation & Laparoscopic Surgery, Digestive & Vascular Surgery Center, The First Affiliated Hospital of Xinjiang Medical University, Urumqi, China; 2 State Key Laboratory of Pathogenesis, Prevention, Treatment of High Incidence Diseases in Central Asia, Xinjiang Medical University, Urumqi, China; 3 Department of Hepatobiliary & Hydatid Disease, Digestive & Vascular Surgery Center, The First Affiliated Hospital of Xinjiang Medical University, Urumqi, China

**Keywords:** Chagas heart disease, meta analysis, organ failure, survival, transplantation

## Abstract

**Introduction:**

Chagas heart disease (ChHD) is a leading trigger of end-stage heart failure in Latin America, with heart transplantation as its final treatment. Large-scale individual patient data (IPD) evidence on transplant immunosuppression, parasite reactivation and long-term prognosis remains scarce.

**Methods:**

We conducted an IPD meta-analysis by retrieving eligible studies up to October 4, 2025, extracting individual clinical data and adopting one-stage IPD and Cox regression analyses.

**Results:**

Sixteen studies with 305 ChHD transplant recipients were enrolled. Male sex was associated with increased risks of complications, reactivation, and death in observational analyses. Similarly, AZA exposure was associated with a lower risk of reactivation, and MMF exposure was associated with a lower risk of death. Notably, exploratory historical comparison suggests relatively favourable long-term survival among selected ChHD transplant recipients relative to published cohorts of other cardiomyopathies, though this finding cannot be adequately adjusted for key patient-level confounders.

**Discussion:**

These associations are subject to confounding by indication, transplantation era, monitoring strategies, and switching-time bias. They therefore cannot be interpreted as causal treatment effects, nor can they establish that any one regimen is superior. In selected patients with end-stage Chagas heart disease, heart transplantation is a feasible treatment option associated with acceptable long-term survival outcomes.

**Systematic Review Registration:**

PROSPERO [CRD420251163696].

## 1 Introduction

ChHD, also termed American trypanosomiasis, is a chronic parasitic infection caused by the protozoan *Trypanosoma cruzi* (T. cruzi) (1). Endemic primarily in tropical and subtropical regions of Latin America, it represents a major neglected tropical disease and a persistent public health challenge in the region (2). Transmission occurs predominantly via the bite of infected triatomine bugs, as well as through blood transfusion, mother-to-child transmission and organ transplantation. The infection is highly insidious, with most early cases being asymptomatic and thus prone to underdiagnosis or misdiagnosis (3, 4). According to WHO estimates, several million people worldwide are chronically infected with T. cruzi. Among those who do not receive timely intervention, approximately one-third will progress to irreversible cardiac involvement—ChHD—the most severe and lethal manifestation of the disease (5, 6).

ChHD caused by T. cruzi infection, represents a leading cause of heart failure and sudden cardiac death in Latin America, constituting a major indication for transplantation in this region (7). Recipients with pre-existing T. cruzi infection face a high risk of parasite reactivation from a latent state following heart transplantation, driven by potent immunosuppressive therapy (8). Reactivation of acute Chagas disease manifests with diverse and nonspecific clinical presentations, ranging from fever and cutaneous lesions to severe myocarditis or even more devastating meningoencephalitis (9). This complication poses a significant diagnostic and therapeutic challenge, contributing substantially to post-transplant morbidity and mortality. Consequently, the principal challenge in managing recipients of heart transplants for ChHD is to effectively prevent and promptly identify disease reactivation. This requires an integrated approach encompassing prophylactic antitrypanosomal therapy, stringent laboratory and clinical surveillance protocols, and optimized immunosuppressive regimens (10). Nevertheless, substantial heterogeneity exists across centers regarding these critical aspects. A consensus is lacking on multiple fronts: the optimal duration and efficacy of prophylactic therapy, the precise incidence and associated risk factors for reactivation, and the most effective immunosuppressive regimen.

The management of infection-related organ failure in solid organ transplant recipients presents a fundamental challenge: clinicians must balance the intensive immunosuppression required to prevent graft rejection against the need to preserve sufficient immunocompetence to contain latent infections. To address this, we focused on heart transplantation for ChHD as a model for T. cruzi infection. Through a systematic review and meta-analysis, we aimed to characterize long-term survival, complication profiles and infection reactivation rates, and to explore the influence of immunomodulatory strategies on clinical outcomes.

## 2 Methods

### 2.1 Search strategy and selection criteria

This systematic review incorporates an IPD meta-analysis, adhering to the Preferred Reporting Items for Systematic Reviews and Meta-Analyses of Individual Participant Data (PRISMA-IPD) guidelines. The assessment also includes case reports and case series, as guided by relevant methodological standards for systematic reviews. Currently, the majority of evidence regarding solid organ transplantation in the context of parasitic infections is derived from non-comparative case reports and case series. Consequently, all relevant case reports and case series were collected and synthesized in accordance with established guidelines. The study protocol was registered *a priori* with the International Prospective Register of Systematic Reviews (PROSPERO; registration number CRD420251163696). This systematic review and IPD meta-analysis was conducted in accordance with the PRISMA-IPD reporting guidelines (11).

A comprehensive literature search was conducted across three electronic databases: PubMed, Web of Science Core Collection, and Scopus, from their inception until October 4, 2025, without language restrictions. For each database, a tailored search strategy was developed using a combination of keywords and controlled vocabulary terms related to the two primary concepts: parasitic infections and solid organ transplantation. To mitigate potential publication bias, we also manually searched for relevant conference abstracts within these databases. Furthermore, the reference lists of all included studies and relevant review articles were examined to identify additional potentially eligible records. All retrieved citations were managed using EndNote software. The study selection process involved two phases. First, two reviewers independently screened titles and abstracts against the predefined eligibility criteria. Second, the full texts of potentially relevant records were independently assessed by the same reviewers for final inclusion. In instances of overlapping patient data reported across multiple publications, the publication with the most recent date or the most comprehensive dataset was selected. Any discrepancies between reviewers were resolved through consensus discussion.

Inclusion criteria encompassed studies of any design involving recipients of solid organ transplants performed specifically for end-stage organ failure attributable to a documented parasitic infection. Outcomes assessed included the incidence of post-transplant complications, parasite reactivation, and overall survival. Exclusion criteria were applied to studies reporting: (1) transplantation primarily for non-parasitic etiologies, (2) *de novo* parasitic infections acquired after the transplant procedure, or (3) investigations focused solely on post-transplant screening for parasitic infections without prior infection-related organ failure.

The methodological quality of case reports and case series was assessed using the tool developed by Murad et al. (12). For cohort studies, the Newcastle-Ottawa Scale (NOS) was employed (13). The questions within these instruments were adapted as necessary to ensure relevance to the specific context of this review. For the single open-label clinical trial identified, its methodological quality was evaluated using the same criteria applied to case series (Supplementary Data, pages 14–17).

### 2.2 Data collection

Corresponding authors of eligible studies were contacted to solicit collaboration and request access to IPD. Acquired IPD underwent a process of curation and harmonization. Subsequently, summary statistics derived from the IPD were cross-validated against aggregated data reported in the original publications; this data extraction was performed in duplicate by two independent reviewers. For studies on heart transplantation in the context of T. cruzi infection, data extraction (performed independently by two reviewers) encompassed: country, age, gender, use of antiparasitic therapy, immunosuppressive regimen, incidence of post-transplant complications and parasite reactivation, and duration of the longest follow-up. The definition of parasite reactivation varied across the primary studies. To address this, we predefined standardized harmonization rules: (i) clinical reactivation, defined as the presence of Chagas disease-related clinical manifestations such as fever, skin lesions, myocarditis, or meningoencephalitis, together with any parasitological evidence (positive PCR, microscopic detection of parasites, or detection of protozoa in myocardial biopsy tissue); and (ii) laboratory-defined asymptomatic reactivation, defined as the absence of typical clinical manifestations but with consecutive or single positive peripheral blood PCR results, after excluding persistent pre-transplant baseline positivity. This IPD analysis classified both categories as reactivation events. We defined “post-transplant complications” as a composite endpoint comprising any major adverse event occurring after transplantation, including acute rejection, severe infection, cardiovascular events, bleeding, and surgical complications. Minor, transient laboratory abnormalities that did not require clinical intervention were not included. In the secondary analysis of published survival data, obtaining raw data for each individual patient is critical, as the IPD approach is regarded as the gold standard for data analysis. We employed an easy-to-use two-stage method to reconstruct IPD from published Kaplan–Meier curves: in the first stage, the coordinates of the original data were extracted, and in the second stage, the IPD were reconstructed using the proposed method (14).

### 2.3 Data analysis

A conventional meta-analysis of aggregated data was performed. For dichotomous outcomes, such as postoperative complications and parasite reactivation, event rates were calculated and are presented as proportions (ranging from 0 to 1) with their corresponding 95% CI. We performed a one-stage IPD meta-analysis. Complications and reactivation were binary outcomes and were analyzed using mixed-effects logistic regression. Survival outcomes were analyzed using a shared-frailty Cox proportional hazards model. Among the 305 patients, 150 had complications, 81 had reactivation, and 81 died; immunosuppressive regimen data were complete for 303 patients. Each multivariable model included no more than three predictors, thereby meeting the requirement of at least 10 outcome events per variable. Age was not included in the individual-level multivariable models because most primary studies provided only study-level summary values. Transplantation era, disease severity, country or center, reactivation monitoring strategies, and antiparasitic prophylaxis could not be adequately adjusted for at the individual level; these factors were therefore listed as limitations. Results are reported as odds ratio (OR) and hazard ratios (HRs) with 95% CI. Patient survival was estimated using the Kaplan–Meier method, with 1-, 5-, and 10-year survival rates reported. Differences in survival curves between groups were compared using the log-rank test. A two-sided *P*-value of less than 0.05 was considered statistically significant. All analyses were conducted using GraphPad Prism, Review Manager and IBM SPSS Statistics.

## 3 Results

### 3.1 Study inclusion and quality assessment

#### 3.1.1 Literature search and screening process

A comprehensive search of PubMed, Web of Science, and Scopus databases yielded 9,447 potentially relevant records. After removing 2,869 duplicates using EndNote software, 6,578 unique records remained for title and abstract screening. Following the exclusion of 5,629 records deemed irrelevant, 949 full-text articles were assessed for eligibility. Ultimately, 16 studies meeting the inclusion criteria were selected, providing complete IPD for 305 patients. We contacted the corresponding authors of all 16 eligible studies (Figure 1).

**Figure 1:**
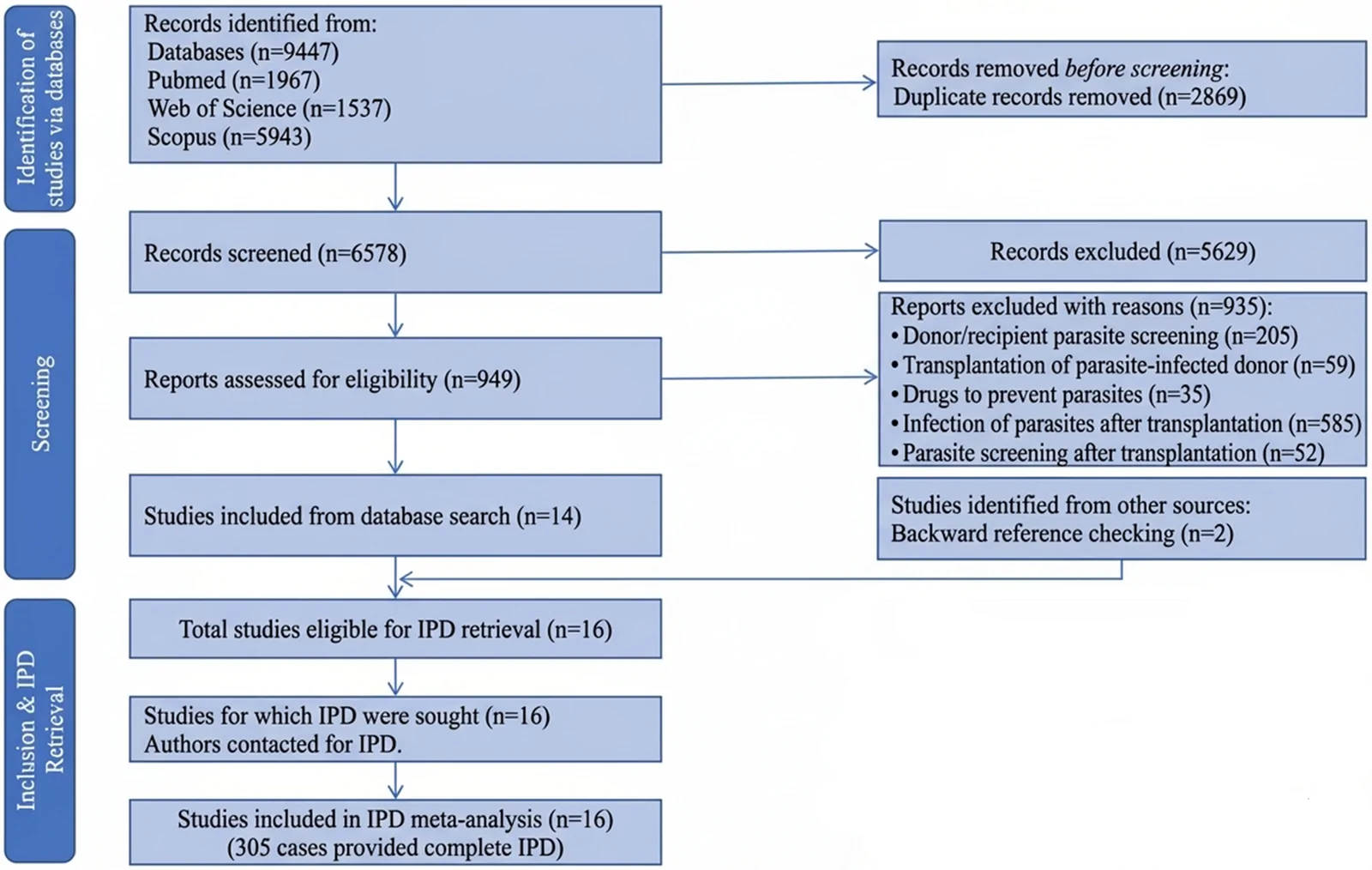
Study selection, including numbers of participants.

#### 3.1.2 Baseline characteristics of included cases

A total of 305 heart transplant recipients with T. cruzi ere included, primarily from Brazil (*n* = 234, 76.7%) and the United States (*n* = 69, 22.6%). The cohort comprised 169 males (55.4%) and 136 females (44.6%). The main immunosuppressive regimens were azathioprine (AZA) (35.7%) and mycophenolate mofetil (MMF) (53.1%), with 10·5% of patients undergoing postoperative regimen adjustment. The observed rates were 49.2% for postoperative complications, 26.6% for disease reactivation, and 73.4% for overall survival (Table 1).

**Table 1:** Characteristics of IPD in heart transplantation of Chagas heart disease (chD) patients.

Study name	Country	Size (*n*)	Age (years)	Gender (male; *n* %)	Pre-HT medication (*n*)	Post-HT medication (*n*)	Immunosuppressive regimen (*n*)	Postoperative complication (*n*)	Reactivation (*n*)	Survival (1–5–10 years) or outcome (*n* %)
Benatti et al. (15)	USA	25	53 ± 7.58	48%	0	0	MMF (14), AZA (11)	6	6	96%–76%–57%
Benvenuti et al. (16)	Brazil	1	36	Male	0	Benznidazole	AZA	1	1	Alive (0–9 years)
Bestetti et al. (17)	Brazil	1	56	Male	0	0	Dressing group	1	1	Death (0–33 years)
Bestetti et al. (18)	Brazil	1	39	Male	0	Benznidazole	MMF	1	1	Alive (1–25 years)
Biro et al. (19)	USA	1	61	Male	0	Benznidazole	MMF	1	1	Alive (0–41 years)
Bocchi and Fiorelli (20)	Brazil	117	39 ± 11	51%	0	Benznidazole (2)	AZA (117)	48	2	62.4%–55.6%–46.2%
Campos et al. (21)	Brazil	17	40 ± 13.8	94%	0	Benznidazole (17)	MMF (6), AZA (11)	17	17	55.6%–44.4%–33.3%
de Oliveira et al. (22)	Brazil	9	54 ± 10.95	45%	0	Benznidazole (2)	MMF (9)	9	2	66.7%-N/A-N/A
Gómez et al. (23)	Colombia	1	59	Male	0	0	N/A	1	1	Death (7 years)
Furquim et al. (24)	Brazil	85	47 ± 11.7	54%	0	Benznidazole (55)	MMF (37), AZA (19), Dressing group (29)	52	20	81.2%–77.6%-N/A
Gray et al. (25)	USA	31	54 ± 9.8	58%	0	Benznidazole (19)	MMF (29), Dressing group (2)	2	19	96.8%-N/A-N/A
Kransdorf et al. (26)	USA	11	55 ± 8.97	55%	0	Benznidazole (4), Nifurtimox (2)	MMF (11)	3	5	81.8%-N/A-N/A
Pinesi et al. (27)	Brazil	1	26	Male	0	Benznidazole	AZA	1	1	Alive (2–4 years)
Riganti et al. (28)	Argentina	1	59	Female	0	Benznidazole	MMF	1	1	Alive (0–16 years)
Ruzza et al. (29)	USA	1	53	Female	0	Benznidazole	Dressing group	1	1	Alive (2 years)
Tomimori-Yamashita et al. (30)	Brazil	2	39 ± 14.85	50%	0	Benznidazole (2)	AZA	2	2	Alive (3–16 years) Alive (1–41 years)

### 3.2 Analysis of prognostic factors

#### 3.2.1 Factors influencing postoperative complications

In logistic regression analyses, male sex was associated with an increased risk of complications (OR = 8.230, 95% CI: 4.93–13.96; *P* < 0.001). Neither AZA vs. MMF (OR = 0.678, 95% CI: 0.47–1.23; *P* = 0.172) nor AZA-to-MMF switching vs. MMF (OR = 1.56, 95% CI: 0.617–3.959; *P* = 0.346) was significantly associated with complications (Table 2).

**Table 2:** Univariable one-stage IPD meta-analysis of factors associated with complication, reactivation, and survival in Chagas heart disease (ChHD) patients.

Variable	Compare	Complication	*p* value
		Logistic model	
		OR, 95%CI	
Gender	Male vs. Female	8.230 (4.93, 13.96)	<0.001
Immunosuppressive therapy	AZA vs. MMF	0.678 (0.47, 1.23)	0.172
	AZA to MMF vs. MMF	1.56 (0.617, 3.959)	0.346
		Reactivation	*p* value
		Logistic model	
		OR, 95%CI	
Gender	Male vs. Female	5.035 (2.626, 9.655)	<0.001
Immunosuppressive therapy	AZA vs. MMF	0.121 (0.063, 0.233)	<0.001
	AZA to MMF vs. MMF	0.385 (0.157, 0.943)	0.037
Variable		Survival	*p* value
		Shared frailty cox model	
		HR, 95%CI	
Gender	Male vs. Female	11.648 (5.906, 22.973)	<0.001
Immunosuppressive therapy	AZA vs. MMF	2.903 (1.724, 4.888)	<0.001
	AZA to MMF vs. MMF	0.346 (0.102, 1.173)	0.089

#### 3.2.2 Factors affecting disease reactivation

In logistic regression analyses, male sex was associated with the outcome compared with female sex (OR = 5.035, 95% CI: 2.626–9.655; *P* < 0.001). AZA exposure was associated with the outcome compared with MMF exposure (OR = 0.121, 95% CI: 0.063–0.233; *P* < 0.001), and AZA-to-MMF switching was associated with the outcome compared with MMF exposure (OR = 0.385, 95% CI: 0.157–0.943; *P* = 0.037). Specifically, AZA-based regimen was observationally associated with a significantly lower risk of reactivation, whereas a MMF-based regimen was observationally associated with a significantly reduced mortality risk. These observational associations may be subject to confounding and do not establish causal treatment effects (Table 2).

#### 3.2.3 Factors influencing survival outcomes

In shared-frailty Cox models, male sex was associated with the outcome compared with female sex (HR = 11.648, 95% CI: 5.906–22.973; *P* < 0.001). AZA exposure was associated with the outcome compared with MMF exposure (HR = 2.903, 95% CI: 1.724–4.888; *P* < 0.001), whereas AZA-to-MMF switching was not significantly associated with the outcome compared with MMF exposure (HR = 0.346, 95% CI: 0.102–1.173; *P* = 0.089) (Table 2).

#### 3.2.4 Survival curve analysis

The results demonstrated a statistically significant difference in survival among patients receiving different immunosuppressive regimens (Log-rank *P* < 0.0003). Furthermore, patients who experienced complications exhibited markedly lower survival compared to those without complications (HR = 11.77, Log-rank *P* < 0.0003), and similarly, survival was significantly reduced in patients with disease reactivation relative to those without reactivation (HR = 11.77, Log-rank *P* < 0.0388) (Figures 3A,B).

### 3.3 Comparative survival analysis

#### 3.3.1 ChHD vs. other cardiomyopathies in heart transplantation

This study did not include a contemporaneous parallel control group. The survival comparison was therefore conducted as an exploratory analysis using external historical cohort data from published studies. External historical data show that heart transplant recipients with ChHD exhibited significantly superior long-term survival compared to those with IDC or ischemic cardiomyopathy. The 1-, 5-, and 10-year survival rates for ChHD patients were 91%, 72%, and 65%, respectively. In contrast, the corresponding rates were 83%, 51%, and 35% for IDC patients, and 78%, 42%, and 28% for patients with ischemic cardiomyopathy (20). This difference was statistically significant (Log-rank *P* < 0.0001). Survival curves demonstrated a gradual decline in the ChHD group, whereas both the IDC and ischemic cardiomyopathy groups showed a pronounced drop in survival within 1–3 years post-transplant, with the survival gap widening over time (Figure 3C).

## 4 Discussion

Chronic parasitic infections, if left to progress over time, inevitably precipitate irreversible end-stage organ failure. For such patients, Solid organ transplantation is a life-saving treatment that can prolong survival in patients with end-stage disease and represents a critical form of life support for these patients. Heart transplantation replaces irreversibly damaged myocardial tissue and can thereby interrupt the pathological process of ongoing myocardial injury, but it cannot eradicate latent Trypanosoma cruzi infection or cure Chagas disease itself (31). Drawing on individual patient data from 305 patients with T.cruzi-induced ChHD who underwent heart transplantation across 16 clinical studies, this observational IPD analysis shows that selected patients with end-stage ChHD can achieve acceptable long-term survival after heart transplantation. However, because no contemporaneous matched non-transplant control group was available, it cannot be established that transplantation itself confers a causal survival benefit. In the present cohort, overall survival was 73.4%, postoperative complications occurred in 49.2% of recipients (Overall survival was defined as the proportion of patients in this cohort who were still alive at their respective last follow-up), and T. cruzi reactivation was observed in 26.6%. By stark contrast, longitudinal follow-up data indicate that patients with advanced ChHD who do not undergo transplantation have a 5-year survival of less than 40% (32), with the majority ultimately succumbing to progressive refractory heart failure, malignant ventricular arrhythmias, systemic parasitic dissemination, or central nervous system involvement by T. cruzi (33). This IPD meta-analysis suggests that carefully selected patients with end-stage Chagas heart disease who undergo heart transplantation can achieve acceptable long-term survival outcomes. However, this study did not include a contemporaneous matched control group of transplant candidates who did not undergo transplantation. The comparison with survival data from non-transplanted patients in external studies is therefore an indirect reference only and is insufficient to establish a causal survival benefit of heart transplantation. Nevertheless, it supports the important clinical value of this treatment for this disease.

From a pathophysiological standpoint, chronic T. cruzi infection follows a self-perpetuating disease trajectory: the parasite invades cardiomyocytes, establishes long-term latency, and perpetuates local chronic inflammatory infiltration, culminating in extensive myocardial fibrotic remodelling, progressive ventricular destruction, and ultimately end-stage heart failure. Heart transplantation abrogates this pathological cascade by surgically replacing all irreversibly damaged myocardium, thereby constituting a curative strategy that halts disease progression at its root (34, 35). However, unlike transplantation for non-infectious aetiologies such as ischaemic or idiopathic cardiomyopathy, ChHD recipients face an inherent and fundamental clinical paradox: the potent immunosuppression required to avert graft rejection inevitably compromises immune surveillance against latent T. cruzi reservoirs in the myocardium, viscera and brain, thereby heightening the risk of postoperative parasitic reactivation (22). The 26.6% rate of T. cruzi reactivation observed in this large, multicentre individual-patient cohort graphically illustrates the unique clinical dilemma inherent to transplantation for parasitic disease. Our survival analysis further revealed that despite achieving acceptable long-term survival following comprehensive management—including intensified antiparasitic therapy and judicious immunosuppression tapering—patients who experienced T. cruzi reactivation had a significantly poorer overall prognosis than those who remained reactivation-free throughout follow-up (HR = 1.712, log-rank *P* = 0.0388) (Figure 3B).

In the present cohort of heart transplant recipients with ChHD, male sex was associated with increased risks of complications, reactivation, and death. Male sex was associated with an increased risk of postoperative complications (HR = 5.256, Log-rank *P* < 0.0001), disease reactivation (HR = 5.544, Log-rank *P* < 0.0001), and mortality (HR = 5.948, Log-rank *P* < 0.0001) (Figures 2A–C), conferring a significantly worse prognosis compared to female recipients. The mechanisms underlying this sex-based disparity are likely multifactorial and complex. Historical research shows that primarily in murine models, indicate that male mice exhibit greater susceptibility to T. cruzi infection and higher associated mortality compared to females. Furthermore, in certain genetically defined mouse models, males display increased vulnerability to acute infection, whereas females demonstrate a more effective control of parasitemia (36). Moreover, hormones such as estrogen have been shown to reduce mortality in T. cruzi -infected mice, potentially through the modulation of macrophage activity (37). Research by Prado et al. demonstrated that gonadectomy altered the progression of T. cruzi in female mice. Specifically, ovariectomized animals exhibited higher parasitemia levels compared to both control and sham-operated groups, suggesting that sex hormones may modulate innate immune responses (38, 39). A similar influence of sex on susceptibility to T. cruzi infection has also been documented in human populations (40, 41).

**Figure 2:**
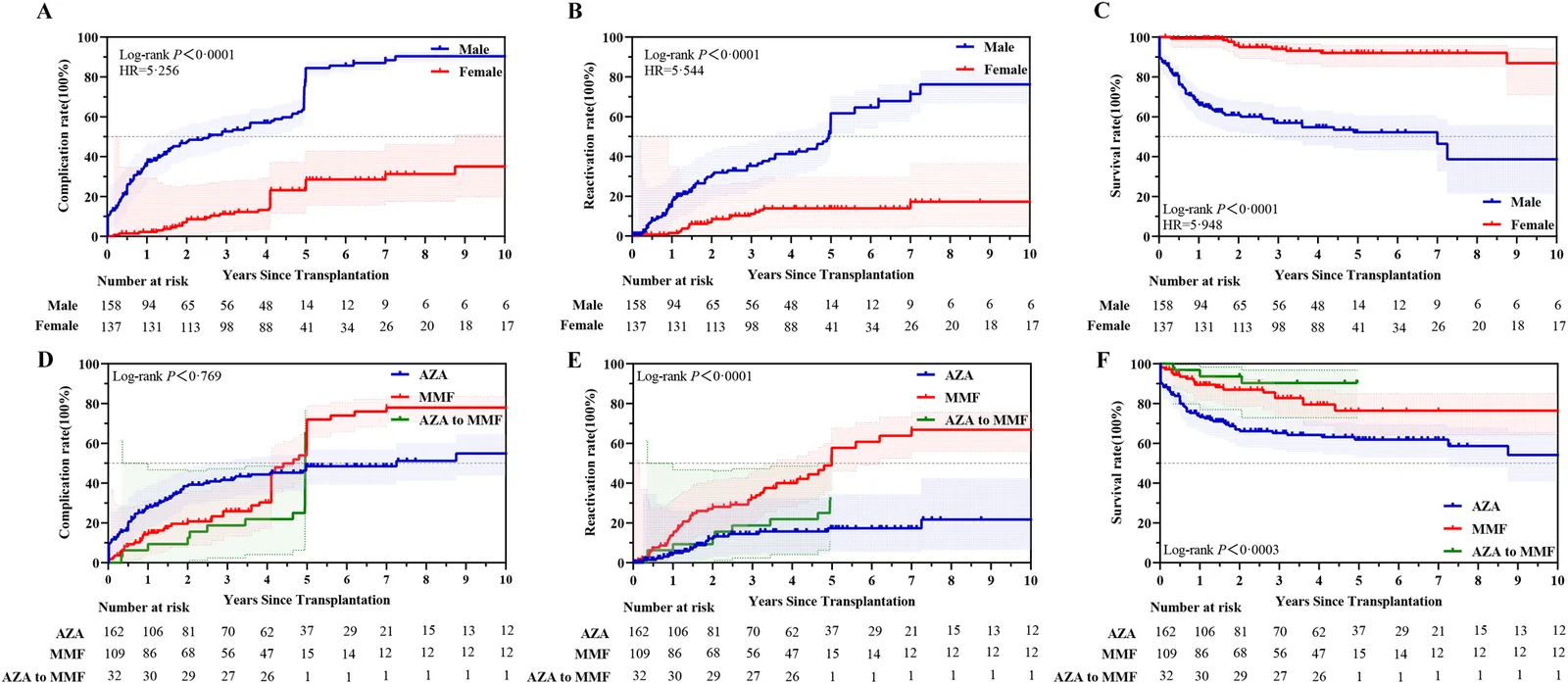
Factors affecting complication reactivation and overall survival after heart transplantation in Chagas heart disease patients. **(A)** The relationship between gender and complications. Log-rank test *P* = 0.0001; HR = 5.256. **(B)** The relationship between gender and reactivation. Log-rank test *P* = 0.0001; HR = 5.544. **(C)** The relationship between gender and overall survival. Log-rank test *P* = 0.0001; HR = 5.948. **(D)** The relationship between immunosuppressive regimen and complications. Log-rank test *P* = 0.769. **(E)** The relationship between immunosuppressive regimen and reactivation. Log-rank test *P* = 0.0001. **(F)** The relationship between immunosuppressive regimen and overall survival. Log-rank test *P* = 0.0003.

Analysis of clinical outcomes revealed that the choice of immunosuppressive regimen influenced both the risk of disease reactivation (Log-rank *P* < 0.0001) and patient survival (Log-rank *P* < 0.0003) following heart transplantation for T. cruzi infection (Figures 2E,F). This study identified two associations in opposite directions. When exposure was classified according to the regimen received at the time of the event, AZA exposure was associated with a lower risk of T. cruzi reactivation, whereas MMF exposure, including having switched to MMF by the time of the event, was associated with better survival. These associations are clinically informative but cannot be interpreted as established mechanisms or treatment effects. The association between AZA exposure and a lower risk of reactivation may involve inhibition of lymphocyte proliferation that alters the parasite microenvironment; however, this mechanism was not directly tested in this study. This association may also be influenced by reactivation monitoring intensity, PCR testing frequency, antiparasitic prophylaxis strategies, center-specific practice, and transplantation era (42, 43). Similarly, the association between MMF exposure and better survival does not establish that MMF acts by reducing T. cruzi-mediated myocardial inflammation. Patient selection, the occurrence and management of rejection, renal function, tolerability, comorbidity burden, and center or era differences may all contribute to confounding (44). The present findings suggest that, when exposure was classified according to the regimen at the time of the event, AZA exposure was associated with a lower risk of Chagas reactivation, whereas MMF exposure, including having switched to MMF by the time of the event, was associated with better survival. However, because the exact date of switching was unavailable, patients who switched from AZA to MMF must have survived long enough to be reclassified into the MMF or post-switch regimen. This association therefore cannot be interpreted as the net benefit of sequential AZA-to-MMF treatment. In particular, in the AZA-to-MMF subgroup, only one patient remained at risk after approximately 5 years, so long-term survival estimates were unstable. On the basis of the available data, we do not make a definitive recommendation for sequential AZA-to-MMF treatment; this strategy should serve only as a hypothesis for future prospective studies.

Exploratory comparisons with external historical cohorts showed that patients with ChHD had 1-, 5-, and 10-year survival rates of 91%, 72%, and 65%, respectively. These estimates were higher than those reported in historical cohorts of patients with idiopathic dilated cardiomyopathy (IDC; 66%, 51%, and 30%) and ischemic cardiomyopathy (ICM; 56%, 41%, and 26%) in an unadjusted comparison (log-rank *P* < 0.0001; Figure 3C). The ChHD survival curve showed a more gradual decline; however, this comparison was unadjusted and was based on external published data with reconstructed survival information rather than a contemporaneous matched control population. These findings are consistent with the previously described “survival paradox” observed in Brazilian multicenter studies of heart transplantation for ChHD (20), but the present analysis cannot confirm that the observed difference is attributable to ChHD etiology or to transplantation itself. Possible explanations proposed previously include a lower burden of generalized vasculopathy and cardiac allograft vasculopathy in ChHD than in ICM, less inherent immune dysregulation than in IDC, and younger age with fewer comorbidities among selected ChHD recipients; however, these remain hypotheses because no contemporaneous matched comparator population was available, and patient-level adjustment for age, comorbidity, transplant era, center, immunosuppressive regimen, and follow-up was not possible. A moderately increased risk of post-transplant malignancy has been reported in some studies, but the present historical comparison cannot determine its relationship to survival. Collectively, these observational findings suggest that ChHD should not be considered an absolute contraindication to heart transplantation and that selected patients may achieve acceptable long-term survival. However, the evidence does not prove that transplantation itself causes the observed survival difference, that any specific immunosuppressive regimen is superior, or that male sex causally worsens outcomes. Prospective studies with contemporaneous controls and standardized reactivation surveillance are needed.

**Figure 3:**
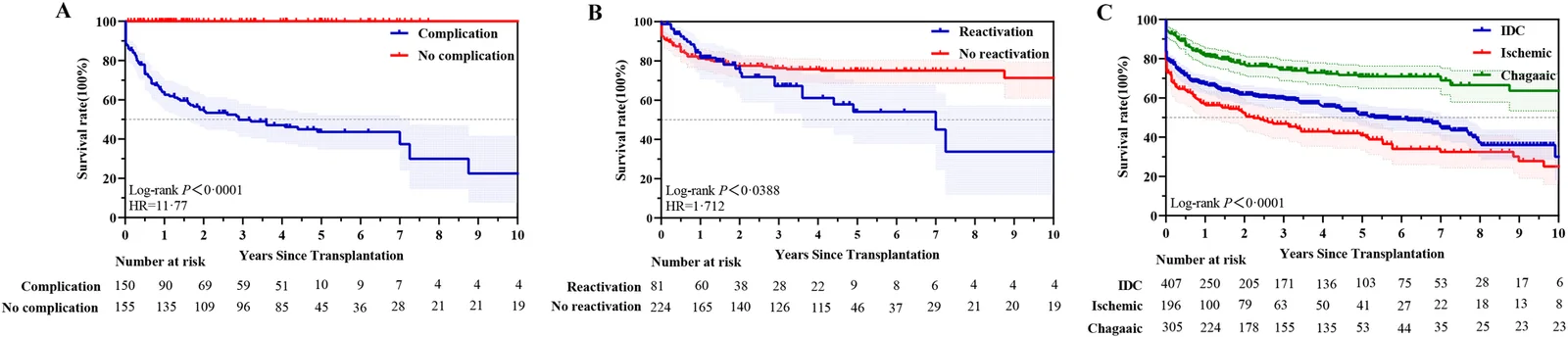
Effect of complications and reactivation on survival rate after heart transplantation in Chagas disease patients. **(A)** The effect of complications on survival rate. Log-rank test *P* = 0·0001; HR = 11·77. **(B)** The effect of reactivation on survival rate. Log-rank test *P* = 0·0388; HR = 1·712. **(C)** Compare the overall survival after cardiac transplantation between Chagas heart disease and IDC and ischemic heart transplantation.

This study has several limitations. First, the evidence base mainly comprised case reports, case series, and retrospective cohort studies, with no prospective randomized controlled trials, making it susceptible to selection, reporting, and publication biases, and residual confounding. Second, despite requesting IPD from original authors, data on age, antiparasitic treatment, immunosuppressive switching, reactivation monitoring, and some event times remained missing, reducing estimate precision and limiting confounder adjustment. Third, only heart transplant recipients with ChHD were included, limiting generalizability to other rare parasitic infections.

Fourth, immunosuppressive regimens were not randomly assigned; AZA vs. MMF choice was influenced by transplantation era, center protocols, rejection risk, comorbidities, and clinical status, causing confounding by indication and era bias. Most studies lacked exact switching dates, so immortal-time and survivor bias could not be fully excluded, and the AZA-to-MMF subgroup had only one patient at risk after about 5 years, making long-term estimates unstable. These results are descriptive only and cannot support recommending sequential AZA-to-MMF treatment.

Fifth, reactivation definitions and monitoring varied across studies, and diagnostic heterogeneity may have affected the pooled reactivation rate of 26.6%; death was not treated as a competing event, so Kaplan–Meier analysis may overestimate cumulative reactivation risk. Post-transplant complications were a composite endpoint with inconsistent items, causing measurement bias. Sixth, male sex risk estimates were relatively large, but models did not fully adjust for age, transplantation era, disease severity, center, rejection, and antiparasitic prophylaxis; male sex cannot be regarded as an established causal independent prognostic factor. Comparisons with IDC, ischemic cardiomyopathy, and non-transplant ChHD cohorts used external historical data rather than contemporaneous controls, so they are exploratory only and cannot establish that ChHD etiology or transplantation itself confers a survival advantage. Heart transplantation cannot clear latent T. cruzi and is not a cure for Chagas disease.

Finally, included studies were highly heterogeneous in follow-up, era, diagnostic procedures, antiparasitic treatment, and immunosuppressive practice, and some had very small samples; study-specific estimates, sensitivity analyses, subgroup analyses, and publication bias assessment were therefore difficult to perform reliably. Quality weighting was not applied, and models became unstable after excluding low-quality studies, so pooled ORs and HRs should be interpreted cautiously. Individual-level data on antiparasitic treatment timing, duration, drug type, and prophylactic vs. rescue use were largely missing, possibly contributing to residual confounding. In summary, findings regarding AZA, MMF, male sex, and the survival advantage of transplantation should be interpreted as observational associations, not causal treatment effects. Future prospective studies with contemporaneous controls, standardized reactivation monitoring, and complete antiparasitic treatment data are needed to validate these findings.

## Data Availability

The original contributions presented in the study are included in the article/Supplementary Material, further inquiries can be directed to the corresponding authors.
