# Supplementary material for: Survival and reactivation risk after heart transplantation in Chagas heart disease: a systematic review and individual patient data meta-analysis
Source: Front Cardiovasc Med. 2026 Oct 28;13:1950776. doi: 10.3389/fcvm.2026.1950776 (PMC13752156; doi:10.3389/fcvm.2026.1950776)
Supplement: Supplementary file 1 [file Supplementaryfile1.docx]

**Supplementary data**

**Content**

**1 Search Strategy .........................................................................................................1**

**2 Data Screening Flow Chart ......................................................................................3**

**3 Statistical Methods ....................................................................................................5**

**4 Full Methodology for Quality Assessment................................................................7**

**5 IPDfromKM: Reconstruct Individual Patient Data (IPD) From Kaplan-Meier Survival Curve...........................................................................................................................14**

**6 Tables of Included Studies......................................................................................32**

**7 Supplementary References.....................................................................................33**

**1 Search Strategy**

**Search Strategy for PubMed:**

(Parasites[Mesh] OR Helminths[Title/Abstract] OR Schistosoma[Title/Abstract] OR Plasmodium[Title/Abstract] OR Malaria[Title/Abstract] OR Trypanosoma[Title/Abstract] OR Leishmania[Title/Abstract] OR Toxoplasma[Title/Abstract] OR Echinococcus[Title/Abstract] OR Ascaris[Title/Abstract] OR Ancylostoma[Title/Abstract] OR Necator[Title/Abstract] OR Trichuris[Title/Abstract] OR Wuchereria[Title/Abstract] OR Strongyloides[Title/Abstract] OR Cryptosporidi [Title/Abstract]) AND (Transplantation[Mesh] OR Grafts[Title/Abstract] OR Graft[Title/Abstract] OR Allograft*[Title/Abstract] OR Autotransplantation*[Title/Abstract] OR Organ transplant*[Title/Abstract] OR Tissue transplant*[Title/Abstract] OR Cell transplant*[Title/Abstract] OR transplant*[Title/Abstract] OR Graft recipient*[Title/Abstract] OR Graft survival*[Title/Abstract] OR Graft rejection*[Title/Abstract] OR transplant recipient*[Title/Abstract])

**Search Strategy for Web of Science:**

(TS=(parasites*) OR AB=(helminths* OR schistosoma* OR plasmodi* OR malaria* OR trypanosoma* OR leishmania* OR toxoplasma* OR echinococcus* OR ascaris* OR hookworm OR trichur* OR filaria* OR strongyloid* OR enterobi* OR giardi* OR cryptosporidi*)) AND (TS=(transplantation*) OR AB=(graft* OR allograft* OR allotransplant* OR Autotransplantation* OR Organ transplant* OR transplant* OR tissue transplant* OR cell transplant* OR Graft recipient* OR Graft survival* OR Graft rejection* OR transplant recipient*))

**Search Strategy for SCOPUS:**

( TITLE-ABS-KEY ( parasites* OR helminths* OR schistosoma* OR plasmodium* OR malaria OR trypanosoma* OR leishmania* OR toxoplasm* OR echinococcus* OR ascaris* OR hookworm OR trichur* OR filaria* OR strongyloid* OR enterobi* OR giardi* OR cryptosporidi* ) ) AND ( TITLE-ABS-KEY ( transplantation* OR graft* OR allograft* OR allotransplant* OR autotransplantation OR "organ transplant" OR "tissue transplant" OR "cell transplant" OR "transplant recipient" OR "graft recipient" OR "graft survival" OR "graft rejection" OR “transplant recipient”)

**2 Data Screening Flow Chart**

**Figure 1: Study selection, including numbers of participants**

PRISMA flow chart adapted for IPD meta-analyses. Case definitions are in the panel. IPD=individual participant data.

Identification

**3 Statistical Methods**

**3.1 Data Integration and Preprocessing**

**3.1.1 Data Source**

A systematic literature search was conducted in three electronic databases: PubMed, Web of Science Core Collection, and Scopus, covering records from their inception until October 4, 2025. Supplementary searches were performed through backward citation tracking of relevant publications. The search strategy aimed to identify case reports, case series, and retrospective cohort studies relevant to the research question.

**3.1.2 Data Extraction and Quality Control**

IPD and aggregated outcome measures were extracted. This included patient baseline characteristics (e.g., age, gender), specifics of the immunosuppressive regimen, and core outcomes such as survival rates and complication incidences. To address missing data, corresponding authors of the original studies were contacted. All extracted data underwent verification for internal consistency and completeness to ensure the integrity of the final IPD dataset.

**3.1.3 Data Standardization**

To enable pooled analysis across heterogeneous studies, key variables (e.g., categories of immunosuppressive agents, definitions of outcome events) were harmonized. This normalization process ensured methodological consistency and comparability of data from different sources.

**3.2 Descriptive Statistical Analysis**

**3.2.1 Description of Baseline Characteristics**

Categorical variables, including gender and immunosuppressive regimen, are presented as frequencies with percentages. Continuous variables, such as age is summarized using the mean.

**3.2.2 Summary of Outcome Measures**

Key outcome measures, including the incidence of postoperative complications, rates of parasite reactivation, and overall survival rates, were calculated and are reported as proportions with corresponding 95% confidence intervals (95% CI).

**3.3 Intergroup Comparisons and Survival Analysis**

**3.3.1 Survival Curve Estimation**

Survival probabilities at 1, 5, and 10 years for patients undergoing heart transplantation for TC infection were estimated using the Kaplan-Meier method. Corresponding survival curves were generated to visually depict the survival experience over time.

**3.3.2 Comparison of Survival Between Groups**

Differences in survival across predefined subgroups were assessed using the log-rank test. Furthermore, this test was employed to compare survival between heart transplant recipients with ChHD due to TC infection versus those with IDC or ischemic cardiomyopathy.

**3.3 Analysis of Prognostic Factors**

Univariable and multivariable Cox proportional hazards regression models were employed to assess the impact of covariates on postoperative complication rates, parasite reactivation rates, and survival outcomes. Hazard ratios (HRs) with corresponding 95% CI were calculated to identify independent prognostic factors associated with these endpoints.

**4 Full Methodology for Quality Assessment**

For this study, the reliability of the analysis hinges on the precise documentation of several key elements. such as allograft rejection and parasite reactivation. Consequently, the completeness of reporting for all these variables was incorporated into the quality assessment framework. Furthermore, given that concurrent or subsequent non-core interventions administered alongside the primary antiparasitic/immunosuppressive regimens could directly influence the outcomes of interest, their reporting was also evaluated. To ensure sufficient observation time for the evaluation of these endpoints, a minimum follow-up duration of 12 months was established a priori for all included studies.

Several included studies exhibited incomplete reporting for various variables. Corresponding authors were contacted via email to request missing data whenever possible. The methodological quality of case reports and case series was assessed using the tool developed by Murad et al., while the Newcastle-Ottawa Scale (NOS) was applied to cohort studies. The items within these instruments were adapted as necessary to ensure relevance to the context of this review. Quality assessment for case reports/series employed an eight-item checklist, with each item assigned one point. Based on the total score, studies were categorized as high quality (7-8 points), moderate quality (4-6 points), or low quality (1-3 points)

Included studies consisted of 11 case reports/case series and 5 retrospective cohorts. Initial quality ratings: case reports/case series (2 high, 9 moderate, 0 low); retrospective cohorts (2 high, 2 moderate, 1 low). Low-quality studies were limited by missing outcomes, short follow-up, and unadjusted confounders.

**Case report / case series study evaluation methods**

Sample Size

1.Does the patient(s) represent(s) the whole experience of the investigator (center) or is the selection method unclear to the extent that other patients with similar presentation may not have been reported?

(Judgement, Unclear=?, Yes=1, No=0)

2. Was the exposure adequately ascertained? (Reporting all parameters of anti-parasitic drug and immunosuppressant regimens, dosages, and treatment cycles for all cases)

3. Was the outcome adequately ascertained? (Reporting graft rejection and parasite recurrence/reactivation status, including occurrence time, diagnostic basis, and treatment outcomes for all cases)

4. Was there a dose–response effect? (Reporting and determining the interval between anti-parasitic drug withdrawal/immunosuppressant adjustment and outcome occurrence)

5. Was follow-up long enough for outcomes to occur? (Following up patients for at least 12 months, with clear reporting of lost-to-follow-up cases and reasons)

6. Were the baseline characteristics of the patients sufficiently reported? (Including age, gender, comorbidities, and disease severity indicators for all cases to ensure comparability)

7. Was the surgical procedure and perioperative management adequately described? (Reporting transplant type, surgical approach, and perioperative supportive treatment details for all cases)

8. Were the criteria for defining outcomes (rejection, recurrence, survival) clearly stated? (Using unified, objective diagnostic standards for outcome assessment, with consistency verification by independent reviewers)

**Case report / series Quality Assessment**

| **Study Author and Year** | **Patient representation/selection method clarity** | **Adequate exposure ascertainment** | **Adequate outcome ascertainment** | **Dose-response effect** | **Sufficient follow-up time** | **Sufficient baseline characteristics reporting** | **Adequate surgical/perioperative description** | **Clear outcome definition criteria** | **Total Score** |
| --- | --- | --- | --- | --- | --- | --- | --- | --- | --- |
| Benvenuti L A 2005 | Unclear | Yes | Yes | Unclear | Yes | Yes | Yes | Yes | 6 |
| Bestetti R B 2004 | Unclear | No | Yes | Unclear | Yes | Yes | Yes | Yes | 5 |
| Bestetti R B 2013 | Unclear | Yes | Yes | Unclear | Yes | Yes | Yes | Yes | 6 |
| Biro M 2023 | Unclear | Yes | Yes | Unclear | No | Yes | Yes | Yes | 5 |
| de Oliveira J F 2025 | Yes | Yes | Yes | Yes | Yes | Yes | Yes | Yes | 8 |
| Fernando G P C 2014 | Unclear | No | Yes | Unclear | Yes | Yes | Yes | Yes | 5 |
| Kransdorf E P 2013 | Unclear | Yes | Yes | Unclear | Yes | Yes | Yes | Yes | 6 |
| Pinesi H T 2019 | Unclear | Yes | Yes | Unclear | Yes | Yes | Yes | Yes | 6 |
| Riganti J 2012 | Unclear | No | Yes | Unclear | No | Yes | Yes | Yes | 4 |
| Ruzza A 2016 | Yes | Yes | Yes | Unclear | Yes | Yes | Yes | Yes | 7 |
| TomimoriYamashita J 1997 | Unclear | No | Yes | Unclear | Yes | Yes | Yes | Yes | 5 |

**Assessment by study**

### **Risk of Bias Assessment**

For the studies included on heart transplantation for Trypanosoma cruzi infection, a risk of bias (RoB) assessment was conducted. This evaluation focused on five core domains: bias arising from the randomization process, bias due to deviations from intended interventions, bias due to missing outcome data, bias in measurement of the outcome, and bias in selection of the reported result. An overall RoB judgment was synthesized, providing a critical foundation for interpreting the reliability of the findings and grading the evidence. The characteristics, causes, and implications of the RoB in each domain are discussed below.

**Bias Arising from the Randomization Process**

The majority of the 16 included studies were case reports/series and retrospective cohort studies, which are non-randomized by design and lack a formal randomization procedure to ensure baseline comparability. Consequently, most were judged as having an unclear risk of bias. A minority of retrospective cohort studies employed methods such as propensity score matching or stratified analysis to adjust for confounders, minimizing selection bias and warranting a low-risk rating. Overall, the absence of high-risk ratings indicates that, while formal randomization was absent, no overt selective allocation bias was introduced that would severely distort the results.

**Bias Due to Deviations from Intended Interventions**

This domain also exhibited a low to unclear risk profile, with no high-risk studies. The interventions under study— heart transplantation for specific parasitic infections—are guided by established clinical protocols regarding surgical techniques, antiparasitic regimens, and immunosuppressive therapy. The core interventions were therefore consistently applied. An unclear risk rating was assigned to some studies due to incomplete reporting of perioperative adjunctive treatments. However, as the implementation of the primary interventions was not compromised, the overall risk in this domain was deemed manageable, preserving the validity of the causal inference between intervention and outcome.

**Bias Due to Missing Outcome Data**

This domain presented one of the more significant sources of potential bias, with several studies rated as having an unclear or even high risk. Inherent to retrospective designs and challenges in long-term clinical follow-up, some studies had missing outcome data. Examples included undocumented long-term survival status, treatment outcomes following parasite recurrence, and detailed records of postoperative complications. Crucially, these studies often failed to describe the reasons for or the pattern (e.g., missing completely at random vs. not) of the missing data and did not employ appropriate statistical methods for handling missingness. Such missing data can lead to biased effect estimates, particularly if the missingness is related to the outcome (e.g., patients with poorer prognosis are more likely to be lost to follow-up), potentially overestimating treatment efficacy and underestimating complication or recurrence rates. To mitigate this, we contacted corresponding authors to obtain missing individual participant data (IPD) where possible, and studies with severe data incompleteness were downgraded in quality, thereby reducing the potential impact of this bias on the overall synthesis.

**Bias in Measurement of the Outcome**

The RoB was predominantly low in this domain, with a minority of studies rated as unclear. The core outcomes—graft rejection, parasite reactivation, patient survival, and postoperative complications—were defined by objective, clinically accepted criteria. Graft rejection was confirmed via imaging or histopathology, parasite recurrence via parasitological or imaging tests, and survival through follow-up records. Most studies clearly described their outcome measurement methods and diagnostic criteria. Some employed independent adjudicators to verify outcomes, reducing measurer subjectivity. A few studies received an unclear rating due to unreported quality control measures (e.g., instrument calibration, diagnostic consistency checks). However, there was no evidence of outcome misclassification due to flawed measurement methods, resulting in an overall low risk of bias and ensuring the accuracy and reliability of outcome ascertainment.

**Bias in Selection of the Reported Result**

The overall RoB here was low to unclear, with no high-risk studies. All included studies reported both primary (e.g., survival, reactivation, complication rates) and secondary outcomes (e.g., adjustments to immunosuppression, antiparasitic drug tolerance) in accordance with clinical reporting standards. There was no evidence of selective reporting, such as omitting negative results. Some studies were rated as unclear because they did not pre-specify their outcomes or failed to report all planned analyses. Nonetheless, no obvious selective reporting bias was detected, supporting the completeness and objectivity of the reported findings.

**Overall Risk of Bias**

The synthesized overall RoB for the included studies was primarily unclear, with a minority rated as low risk. No study was judged to have a high overall risk. This profile is directly linked to the observational design (case reports/series, retrospective cohorts) of the included evidence, which carries inherent biases (e.g., selection, confounding) not controllable through randomization as in randomized controlled trials (RCTs). Consequently, the overall RoB is inherently higher than that of RCTs. However, through rigorous study selection, independent dual quality assessment, supplementation of missing data, and standardized statistical analysis, we have endeavored to minimize the influence of these biases, thereby enhancing the reliability of the synthesized evidence.

**5 IPDfromKM: Reconstruct IPD From Kaplan-Meier Survival Curve**

Typical information used for meta-analysis of survival data reported from clinical trials often includes a summary of outcomes for each arm, including but not limited to hazard ratios and Kaplan-Meier (K-M) curves along with the number of patients at risk . When applying secondary analysis on such published survival data, difficulties usually come from insufficient details in the reported data, which are often reported using aggregated summary statistics. For example, when conducting the meta-analysis on time-to-event data, it is possible that the proportional hazard ratio assumption may not hold, and alternative measures of the survival difference are needed to avoid bias ^1,2^. In this case, it is of great importance to obtain individual patient data (IPD), with which one can not only perform the standard survival analysis, but also can assess if the assumption of the original method is appropriate in order to decide whether or not alternative methods should be applied. Furthermore, one can undertake additional subgroup analyses not reported in the aggregated data. For this reason, the IPD approach is considered as the gold standard in data analysis.

However, researchers conducting meta-analysis or other secondary analyses may lack access to the IPD, partly due to the confidentiality of clinical data. Therefore, a method that is able to reconstruct IPD from published K-M curves can greatly facilitate secondary analyses on survival data. Several methods have been reported in the literature. The iterative algorithm based on K-M estimation method (referred to as “iKM” hereafter) proposed by Guyot et al^3^. is a classic approach among many proposed. It has been used in various secondary analyses. For example, Satagopan et al^4^. used it to reconstruct time-to-event data from a melanoma data set for evaluating different treatment benefits according to biomarker subgroups. Wei and Royston^5^ developed a STATA function to apply the iKM algorithm with some adaptions and applied it to reconstruct IPD from K-M curves from multiple trials.

Building on existing methods, we propose an easy-to-use two-stage approach to reconstruct individual patient data (IPD) from published Kaplan-Meier (K-M) curves. The first stage extracts the raw data coordinates, and the second stage reconstructs the IPD using our proposed method. To facilitate the application of our method, we have developed an R package named IPDfromKM along with a companion web-based Shiny application. Both the R package and the Shiny app offer an "all-in-one" functionality, allowing users to extract raw data coordinates from published K-M curves, reconstruct IPD from the extracted coordinates, visualize the reconstructed IPD, assess reconstruction accuracy, and perform secondary analyses based on the reconstructed IPD. We demonstrate the R package and Shiny application using K-M curves from published studies. Extensive simulations and real-world data applications show that the method achieves high accuracy and reliability in estimating the number of events, the number of patients at risk, survival probabilities, median survival time, and hazard ratios^6^.

**The original KM curve^7^**

**This panel shows the summary results that assess the accuracy and reproducibility of the modified-iKM algorithm(Chagas)**

**Table Accuracy evaluation**

| Summary statistics | Value |
| --- | --- |
| Root mean square error (RMSE) | 0·013 |
| Mean absolute error | 0·011 |
| Max absolute error | 0·02 |
| Kolmogorov-Smirnov test statistics (p-value) | 0·36(0) |

**This panel shows the K-M curve and cumulative risk based on the reconstructed IPD, reports landmark survival probabilities at pre-specified times, and display survival times at given survival probabilities**

**Table Landmark survival probability, standard error (SE), and 95% condifence interval (CI) given pre-specified time point**

| Time | Survival probability | SE | 95% lower CI | 95% upper CI |
| --- | --- | --- | --- | --- |
| 5 | 0·76 | 0·085 | 0·610 | 0·947 |
| 10 | 0·56 | 0·099 | 0·396 | 0·793 |
| 15 | 0·56 | 0·099 | 0·396 | 0·793 |
| 20 | 0·40 | 0·098 | 0·248 | 0·646 |

**Table Survival time and 95% condifence interval (CI) given pre-specified survival probabilities**

| Survival probability | Survival time | 95% lower CI | 95% upper CI |
| --- | --- | --- | --- |
| 0·75 | 6·096 | 3·694 | 15·808 |
| 0·50 | 15·808 | 7·625 | NA |
| 0·25 | NA | NA | NA |

**The original KM curve^8^**

**This panel shows the summary results that assess the accuracy and reproducibility of the modified-iKM algorithm(AZA)**

**Table Accuracy evaluation**

| Summary statistics | Value |
| --- | --- |
| Root mean square error (RMSE) | 0·008 |
| Mean absolute error | 0·004 |
| Max absolute error | 0·026 |
| Kolmogorov-Smirnov test statistics (p-value) | 0·55(0) |

**This panel shows the K-M curve and cumulative risk based on the reconstructed IPD, reports landmark survival probabilities at pre-specified times, and display survival times at given survival probabilities**

**Table Landmark survival probability, standard error (SE), and 95% condifence interval (CI) given pre-specified time point.**

| Time | Survival probability | SE | 95% lower CI | 95% upper CI |
| --- | --- | --- | --- | --- |
| 1 | 0·8916 | 0·0724 | 0·7604 | 1 |
| 2 | 0·8359 | 0·0867 | 0·6821 | 1 |
| 3 | 0·8359 | 0·0867 | 0·6821 | 1 |
| 4 | 0·8359 | 0·0867 | 0·6821 | 1 |

**The original KM curve^8^**

**This panel shows the summary results that assess the accuracy and reproducibility of the modified-iKM algorithm(MFM)**

**Table Accuracy evaluation.**

| Summary statistics | Value |
| --- | --- |
| Root mean square error (RMSE) | 0·008 |
| Mean absolute error | 0·006 |
| Max absolute error | 0·006 |
| Kolmogorov-Smirnov test statistics (p-value) | 0·24(0·0042) |

**This panel shows the K-M curve and cumulative risk based on the reconstructed IPD, reports landmark survival probabilities at pre-specified times, and display survival times at given survival probabilities.**

**Table Landmark survival probability, standard error (SE), and 95% condifence interval (CI) given pre-specified time point**

| Time | Survival probability | SE | 95% lower CI | 95% upper CI |
| --- | --- | --- | --- | --- |
| 1 | 0·9167 | 0·0461 | 0·8307 | 1 |
| 2 | 0·888 | 0·0528 | 0·7904 | 0·9977 |
| 3 | 0·8307 | 0·063 | 0·7159 | 0·9639 |
| 4 | 0·8307 | 0·063 | 0·7159 | 0·9639 |

**The original KM curve^8^**

**This panel shows the summary results that assess the accuracy and reproducibility of the modified-iKM algorithm(Mudanca)**

**Table Accuracy evaluation.**

| Summary statistics | Value |
| --- | --- |
| Root mean square error (RMSE) | 0·007 |
| Mean absolute error | 0·005 |
| Max absolute error | 0·009 |
| Kolmogorov-Smirnov test statistics (p-value) | 0·56(0) |

**This panel shows the K-M curve and cumulative risk based on the reconstructed IPD, reports landmark survival probabilities at pre-specified times, and display survival times at given survival probabilities**

**Table Landmark survival probability, standard error (SE), and 95% condifence interval (CI) given pre-specified time point**

| Time | Survival probability | SE | 95% lower CI | 95% upper CI |
| --- | --- | --- | --- | --- |
| 1 | 0·9643 | 0·0351 | 0·8979 | 1 |
| 2 | 0·9643 | 0·0351 | 0·8979 | 1 |
| 3 | 0·9286 | 0·0487 | 0·8379 | 1 |
| 4 | 0·9286 | 0·0487 | 0·8379 | 1 |

**The original KM curve^9^**

**This panel shows the summary results that assess the accuracy and reproducibility of the modified-iKM algorithm(chagasic)**

**Table Accuracy evaluation**

| Summary statistics | Value |
| --- | --- |
| Root mean square error (RMSE) | 0·012 |
| Mean absolute error | 0·009 |
| Max absolute error | 0·029 |
| Kolmogorov-Smirnov test statistics (p-value) | 0·33(0) |

**This panel shows the K-M curve and cumulative risk based on the reconstructed IPD, reports landmark survival probabilities at pre-specified times, and display survival times at given survival probabilities**

**Table Landmark survival probability, standard error (SE), and 95% condifence interval (CI) given pre-specified time point**

| Time | Survival probability | SE | 95% lower CI | 95% upper CI |
| --- | --- | --- | --- | --- |
| 2 | 0·613 | 0·0478 | 0·526 | 0·7143 |
| 4 | 0·5724 | 0·0501 | 0·4821 | 0·6796 |
| 6 | 0·5565 | 0·0512 | 0·4647 | 0·6664 |
| 8 | 0·5565 | 0·0512 | 0·4647 | 0·6664 |

**Table Survival time and 95% condifence interval (CI) given pre-specified survival probabilities**

| Survival probability | Survival time | 95% lower CI | 95% upper CI |
| --- | --- | --- | --- |
| 0·75 | 0·503 | 0·0838 | 1·6096 |
| 0·5 | 8·7521 | 2·8335 | NA |
| 0·25 | NA | NA | NA |

**The original KM curve^9^**

**This panel shows the summary results that assess the accuracy and reproducibility of the modified-iKM algorithm(IDC)**

**Table Accuracy evaluation**

| Summary statistics | Value |
| --- | --- |
| Root mean square error (RMSE) | 0·004 |
| Mean absolute error | 0·003 |
| Max absolute error | 0·017 |
| Kolmogorov-Smirnov test statistics (p-value) | 0·19(0) |

**This panel shows the K-M curve and cumulative risk based on the reconstructed IPD, reports landmark survival probabilities at pre-specified times, and display survival times at given survival probabilities.**

**Table Landmark survival probability, standard error (SE), and 95% condifence interval (CI) given pre-specified time point**

| Time | Survival probability | SE | 95% lower CI | 95% upper CI |
| --- | --- | --- | --- | --- |
| 2 | 0·6174 | 0·0247 | 0·571 | 0·6677 |
| 4 | 0·5565 | 0·0263 | 0·5072 | 0·6106 |
| 6 | 0·4928 | 0·0287 | 0·4396 | 0·5524 |
| 8 | 0·3737 | 0·038 | 0·3062 | 0·456 |
| 10 | 0·3003 | 0·0637 | 0·1982 | 0·4551 |
| 12 | 0·3003 | 0·0637 | 0·1982 | 0·4551 |

**Table Survival time and 95% condifence interval (CI) given pre-specified survival probabilities**

| Survival probability | Survival time | 95% lower CI | 95% upper CI |
| --- | --- | --- | --- |
| 0·75 | 0·2856 | 0·0859 | 0·5595 |
| 0·5 | 5·3101 | 4·4182 | 7·6845 |
| 0·25 | NA | NA | NA |

**The original KM curve^9^**

**This panel shows the summary results that assess the accuracy and reproducibility of the modified-iKM algorithm(Ischemic)**

**Table Accuracy evaluation**

| Summary statistics | Value |
| --- | --- |
| Root mean square error (RMSE) | 0·003 |
| Mean absolute error | 0·003 |
| Max absolute error | 0·009 |
| Kolmogorov-Smirnov test statistics (p-value) | 0·08(0·0219) |

**This panel shows the K-M curve and cumulative risk based on the reconstructed IPD, reports landmark survival probabilities at pre-specified times, and display survival times at given survival probabilities**

**Table Landmark survival probability, standard error (SE), and 95% condifence interval (CI) given pre-specified time point**

| Time | Survival probability | SE | 95% lower CI | 95% upper CI |
| --- | --- | --- | --- | --- |
| 2 | 0·5246 | 0·0368 | 0·4572 | 0·602 |
| 4 | 0·4294 | 0·0385 | 0·3602 | 0·512 |
| 6 | 0·3401 | 0·0417 | 0·2675 | 0·4326 |
| 8 | 0·3247 | 0·0426 | 0·2511 | 0·4199 |
| 10 | 0·2505 | 0·0502 | 0·1691 | 0·3711 |
| 12 | 0·2192 | 0·0528 | 0·1367 | 0·3515 |

**Table Survival time and 95% condifence interval (CI) given pre-specified survival probabilities**

| Survival probability | Survival time | 95% lower CI | 95% upper CI |
| --- | --- | --- | --- |
| 0·75 | 0·0223 | 0·001 | 0·173 |
| 0·5 | 2·2305 | 1·0125 | 4·9627 |
| 0·25 | 10·1449 | 8·8513 | NA |

**6 Table of included studies**

**Table Data summary of patients with Chagas heart disease**

|  | **Trypanosoma cruzi** | **Patients,No.(100%)** |  |
| --- | --- | --- | --- |
|  |  | **Heart transplantation** |  |
|  | **Country** |  |  |
|  | Brazil | 234 |  |
|  | USA | 69 |  |
|  | Argentina | 1 |  |
|  | Colombia | 1 |  |
|  | **Gender** |  |  |
|  | Male | 169 |  |
|  | Female | 136 |  |
|  | **Immunosuppressive regimen** |  |  |
|  | AZA | 162 |  |
|  | MMF | 109 |  |
|  | Dressing group | 32 |  |
|  | Unknown | 2 |  |
|  | **Postoperative complication** | 150 |  |
|  | **Reactivation** | 81 |  |
|  | **Survival** | 224 |  |

**Supplementary references**

1.	Earle CC, Pham B, Wells GA. An assessment of methods to combine published survival curves. *Medical decision making : an international journal of the Society for Medical Decision Making*. Jan-Mar 2000;20(1):104-11. doi:10.1177/0272989x0002000113

2.	Lueza B, Rotolo F, Bonastre J, Pignon JP, Michiels S. Bias and precision of methods for estimating the difference in restricted mean survival time from an individual patient data meta-analysis. *BMC medical research methodology*. Mar 29 2016;16:37. doi:10.1186/s12874-016-0137-z

3.	Guyot P, Ades AE, Ouwens MJ, Welton NJ. Enhanced secondary analysis of survival data: reconstructing the data from published Kaplan-Meier survival curves. *BMC medical research methodology*. Feb 1 2012;12:9. doi:10.1186/1471-2288-12-9

4.	Satagopan JM, Iasonos A, Kanik JG. A reconstructed melanoma data set for evaluating differential treatment benefit according to biomarker subgroups. *Data in brief*. Jun 2017;12:667-675. doi:10.1016/j.dib.2017.05.005

5.	Wei Y, Royston P. Reconstructing time-to-event data from published Kaplan-Meier curves. *The Stata journal*. Oct 2017;17(4):786-802.

6.	Liu N, Zhou Y, Lee JJ. IPDfromKM: reconstruct individual patient data from published Kaplan-Meier survival curves. *BMC medical research methodology*. Jun 1 2021;21(1):111. doi:10.1186/s12874-021-01308-8

7.	Benatti RD, Al-Kindi SG, Bacal F, Oliveira GH. Heart transplant outcomes in patients with Chagas cardiomyopathy in the United States. *Clinical transplantation*. Jun 2018;32(6):e13279. doi:10.1111/ctr.13279

8.	Furquim SR, Galbiati LC, Avila MS, et al. Survival of Heart Transplant Patients with Chagas' Disease Under Different Antiproliferative Immunosuppressive Regimens. *Arquivos brasileiros de cardiologia*. Oct 2023;120(10):e20230133. Sobrevida de Pacientes Transplantados Cardíacos com Doença de Chagas Sob Diferentes Regimes de Imunossupressores Antiproliferativos. doi:10.36660/abc.20230133

9.	Bocchi EA, Fiorelli A. The paradox of survival results after heart transplantation for cardiomyopathy caused by Trypanosoma cruzi. First Guidelines Group for Heart Transplantation of the Brazilian Society of Cardiology. *The Annals of thoracic surgery*. Jun 2001;71(6):1833-8. doi:10.1016/s0003-4975(01)02587-5
