# Supplementary material for: Survival and reactivation risk after heart transplantation in Chagas heart disease: a systematic review and individual patient data meta-analysis
Source: Front Cardiovasc Med. 2026 Oct 28;13:1950776. doi: 10.3389/fcvm.2026.1950776 (PMC13752156; doi:10.3389/fcvm.2026.1950776)
Supplement: Supplementary file 2 [file Table1.docx]

**Table S1 Post‑transplant clinical outcomes stratified by gender and immunosuppressive therapy**

| **Gender** | **Number(n)** | **Complication(n%)** | **Reactivation(n%)** | **Death(n%)** |
| --- | --- | --- | --- | --- |
| Female | 136 | 31 (22.8%) | 18 (13.2%) | 10 (7.4%) |
| Male | 169 | 119 (70.4%) | 63 (37.3%) | 71 (42.0%) |
| **Immunosuppressive therapy** | **Number** | **Complication** | **Reactivation** | **Death** |
| AZA | 109 | 56 (51.4%) | 51 (46.8%) | 20 (18.3%) |
| MMF | 162 | 72 (44.4%) | 19 (11.7%) | 57 (35.2%) |
| AZA to MMF | 32 | 21 (65.6%) | 10 (31.2%) | 3 (9.4%) |
